# An investigation of knowledge and attitudes towards antidepressants: a cross-sectional survey of Jordan’s six medical schools

**DOI:** 10.1186/s12888-023-05037-8

**Published:** 2023-08-18

**Authors:** Mahmoud Abdallat, Rand Murshidi, Hana Taha, Dunia Z. Jaber, Muhammad Hammouri, Leen Al-Huneidy, Maram Aljayeh, Hadeel H. Ghanem, Adees Wirtan Bedros, Jaafar Al-Omairi, Rand Abbas, Mustafa Ibrahim Abu-usba, Zaid Alkayed, Radwan Banimustafa, Abdallah Al-Ani

**Affiliations:** 1 Department of Neurosurgery, School of medicine, The University of Jordan, Amman, Jordan; 2 Department of Dermatology, School of medicine, The University of Jordan, Amman, Jordan; 3 Department of Pharmacology, Public Health and Clinical Skills, Faculty of Medicine, The Hashemite University, Zarqa, Jordan; 4 Department of Family and Community Medicine, School of Medicine, The University of Jordan, Amman, Jordan; 5 Department of Neurobiology, Care Sciences and Society, Karolinska Institute, Stockholm, Sweden; 6 School of medicine, The University of Jordan, Amman, Jordan; 7 Department of psychiatry, The University of Jordan, Amman, Jordan; 8 Office of Scientific Affairs and Research, King Hussein Cancer Center, Amman, Jordan

**Keywords:** Antidepressants, Jordan, Medical students, Drug attitude inventory, Knowledge

## Abstract

**Objective:**

Depression is Jordan’s most ravaging mental illness. Despite the growth of antidepressant use, only a handful of studies examine the factors affecting antidepressant knowledge among healthcare workers or medical students. Therefore, we aimed to explore the knowledge and attitudes towards antidepressants across Jordan’s six medical schools.

**Methods:**

Using a cross-sectional design, we investigated the knowledge and attitudes towards antidepressants through the Drug Attitude Inventory and a literature-validated knowledge domain. Clinical students from Jordan’s six medical schools were recruited. Differences in knowledge and attitudes scores were examined by year of study, medical school among other factors. A multivariate linear regression model was utilized to assess predictors of knowledge.

**Results:**

We included a total of 1,234 participants representing Jordan’s six major schools of medicine. About 14.9% of participants had a personal history of antidepressant use while 20.5% reported family history of psychiatric disease. The majority of students demonstrated favorable attitudes towards antidepressants (74.1%). Students demonstrated an average understanding of antidepressants’ mechanism of action, side effects, but not indications in special populations. Senior medical students, higher GPA, higher family income, personal history of antidepressants, and family history of psychiatric illnesses were associated with significantly higher knowledge scores (all p-values < 0.001). In addition to attitudes scores, the aforementioned were positive predictors of knowledge scores in the multivariate model.

**Conclusion:**

Medical students’ knowledge towards antidepressants leaves room for significant improvement. Yet, it is evident that significant differences for both attitudes and knowledge exist across medical schools which may indicate a gap in either training or teaching methodology.

## Introduction

The prevalence of psychiatric disorders has significantly increased over the past few years [1]. Of which, major depression is predicted to be the leading cause of disability in 2023 [2]. Pharmacological interventions, psychotherapy (e.g., cognitive behavioral therapy), or a combination of both are the primary recommended treatment regimens for most variants of depression [3]. Despite their availability and cost effectiveness, less than 44% of patients who meet the criteria for major depression receive appropriate care, while even those who do rarely get the recommended period of four to six months [4, 5]. Of the many elements affecting such a phenomenon, beliefs, concerns, and negative attitudes towards antidepressants have been reported to affect adherence practices [6–8]. Most notably, stigmatizing attributes, especially towards depression, is a major factor contributing to the lower rates of proper care administration [9].

Despite the rising rates of depression and simultaneous growth of antidepressant use, the enduring prejudice and stigma still constitute major obstacles to receiving proper treatment for patients with depression [10–12]. It is important to note that the effect of stigma is more pronounced in traditional societies, which Jordan falls into [13, 14]. Although data regarding psychiatric illnesses in Jordan is scarce, it was shown that the Jordanian population exhibits unfavorable and stigmatizing attitudes towards mental illnesses and their treatments, which may partially explain the lack of demand for mental health services there [15, 16].

Most of the literature conducted on those grounds was primarily concerned with the public’s general perception of depression, with fewer studies focusing on antidepressants [9]. Specifically, limited projects were directed towards the understanding and beliefs held by healthcare professionals, especially medical students [17, 18]. Considering that medical students are at a higher risk of developing depression [19], and are expected to manage and treat cases of depression in their respective populations, investigating their understanding of psychiatric disease and its treatment is of critical importance as it allows for the improvement of provided healthcare.

The interaction between knowledge and attitudes is neither simple nor linear. A variety of theories describing behaviors (i.e., Health Belief Model, Elaboration Likelihood Model) propose that knowledge is an integral variable for the development and alteration of attitudes leading to a probable behavioral change [20]. While some theories postulate that knowledge may mediate the relationship between attitudes and behavior using a continuum model, others imply that a stage-based system is more appropriate [21]. The latter implies that the involvement of different interventions, each with different timings and intensities, is crucial for the development of proper attitudes and behaviors.

In terms of healthcare workers, the literature demonstrates that increasing knowledge improves attitudes, particularly stigma [22]. Also, previous studies emphasize the direct effect of attitudes held by healthcare professionals on altering access and overall quality of care provided to mental health patients [23, 24]. Therefore, we hypothesize that there is an association between attitudes towards and knowledge of antidepressants among future healthcare practitioners. Our study aims to explore the knowledge and attitudes towards antidepressants, examine the effectiveness of medical education in improving them, and model possible factors that may predict knowledge in a group of Jordanian medical students.

## Methods

### Setting, sampling, and design

We conducted this cross-sectional investigation on medical students attending the six Jordanian medical schools (i.e., University of Jordan, Jordan University of Science and Technology, Hashemite University, Mu’tah University, Yarmouk University, and Al Balqa’ Applied University). Data was collected during the period between July 2022 and January 2023. The six major Jordanian medical schools are home to over 5000 medical undergraduates in their clinical years, representing various backgrounds and regions. The doctor of medicine (M.D.) program across Jordan’s six medical schools consists of three years of basic sciences training (i.e., 1st, 2nd, and 3rd ), followed by three years of clinical clerkships and rotations (i.e., 4th, 5th, and 6th ). Medical students in their 4th, 5th, and 6th years (i.e., clinical years) were sampled for inclusion. Participants who refused to participate in the study or did not complete the questionnaire were excluded.

The estimated sample size was calculated using GPower 3.1 and EpiInfo. At a power of 95%, α margin of error of 5%, and an effect size of 30%, a sample of 578 participants was needed to demonstrate statistical differences of appropriate power. Participants were recruited using convenience sampling. Invitations to participate were sent to the official university-affiliated online groups associated with the targeted medical students. Upon agreeing to join, the questionnaire would be facilitated using Google Forms. Participants were limited to a one-time completion of the questionnaire to avoid redundant attempts. This study was approved by the University of Jordan Institutional Review Board and followed the institutional and/or national research committee’s ethical standards and the principles of the World Medical Association’s Declaration of Helsinki.

#### Data collection instrument

The implemented questionnaire was comprised of three domains including (1) sociodemographic characteristics, (2) modified drug attitude inventory (DAI), and (3) knowledge of antidepressants domain. The sociodemographic characteristics domain included a total of 17 items pertaining to gender, year of study, attended university, grade point average (GPA), parents’ educational level, family income, substance misuse (e.g., smoking, alcohol), history of taking antidepressants, and family history of psychiatric illnesses among others. The DAI questionnaire, modified to target antidepressants instead of general medications, is a 10-item metric that was used to measure attitudes of medical students towards antidepressants [25]. The inventory has six positively phrased and four negatively phrased items, each of which is answered on a dichotomous scale (1 = TRUE; -1 = FALSE). A score greater than 0 indicates positive attitudes and vice versa. Knowledge of antidepressants was a self-developed domain based on relevant literature and expert consensus. It is comprised of 23 items across four subdomains including mechanism of action, side effects, consideration for antidepressants use in special groups, and a miscellaneous section. All items were scored using a 5-point Likert scale (1 = Strongly disagree, 2 = Disagree, 3 = Neutral, 4 = Agree, 5 = Strongly agree). After pilot testing on 15 experts and 15 medical students, the domain’s Cronbach’s α was 0.739 and content validity index was 0.837.

### Statistical analysis

Data were analyzed using SPSS version 23. For items utilizing 5-point Likert scales, disagreement responses were grouped together (Strongly disagree and disagree into disagree), while agreement responses were grouped together for ease in reporting (strongly agree and agree into agree). Mean differences within DAI and knowledge domains were compared among different categorizations of participants using the student’s *t*-test and ANOVA. The knowledge of antidepressants score was calculated as the sum of correctly answered items on 3rd domain. A multivariate linear regression model was computed to explore predictors of knowledge of antidepressants. All statistical tests are conducted with a 95% confidence interval and a 5% error margin. A p-value of less than 0.05 is considered statistically significant.

## Results

We included a total of 1,234 participants, with a male-to-female ratio of 1-to-1.4. 5th and 6th year students comprised 54.8% of the entire sample, while 45.2% were 4th year students. The sample was mostly recruited from the University of Jordan (28.7%), followed by the Jordan University of Science and Technology (25.6%). The majority of participants had above average academic standing (75.2%), never smoked (75.7%), nor consumed alcohol (94.5%). Among our sample, 14.9% had a personal experience of using antidepressants, of whom 38.6% had adverse effects. Moreover, 20.5% reported having a family history of psychiatric illnesses. Table 1 demonstrates the characteristics of the included participants.

**Table 1:** Sociodemograhic and psychiatric characteristics of recruited participants

Sociodemographics	N	%
**Gender**		
Male	500	40.5
Female	734	59.5
**Year of study**		
Fourth Year	558	45.2
Fifth Year	346	28.1
Sixth Year	330	26.7
**University**		
Al Balqa Applied University	103	8.3
Hashmite University	184	14.9
Jordan University of Science and Technology	316	25.6
Mu’tah University	120	9.7
University of Jordan	354	28.7
Yarmouk University	157	12.7
**Grade Point Average (GPA)**		
Poor	6	0.5
Fair	19	1.5
Good	281	22.8
Very Good	572	46.4
Excellent	356	28.8
**Mother’s education**		
School	220	17.8
Higher Diploma	264	21.4
Bachelors	561	45.5
Master	121	9.8
PhD	68	5.5
**Father’s education**		
School	194	15.7
Higher Diploma	168	13.6
Bachelors	499	40.4
Master	163	13.2
PhD	210	17
Monthly family income**		
0–149	14	1.1
150–499	143	11.6
500–999	412	33.4
1000+	665	53.9
**Substance misuse and psychological characteristics**	N	(%)
**Smoking history**		
Never	934	75.7
Ex-smoker	104	8.4
Active	196	15.9
**Alcohol consumption history**		
Never	1166	94.5
Sometimes	61	4.9
Regularly	7	0.6
**History of antidepressant use**		
No	1050	85.1
Yes	184	14.9
Compliance to antidepressants*!		
No	33	17.9
Yes	148	80.4
History of adverse effects due to antidepressant use*!		
No	107	58.2
Yes	71	38.6
**History of psychotherapy**		
No	1102	89.3
Yes	132	10.7
**Family history of psychiatric disease**		
No	981	79.5
Yes	253	20.5

*Percentages for the following items are calculated from the number of participants utilizing antidepressants (n = 184).

!Percentages may not add up to 100% due to missing responses.

**Income is reported in Jordanian Dinars (JOD). 1 JOD = 1 U.S Dollars

In terms of attitudes, 74.1% of students demonstrated favorable positions towards the use of antidepressants. The greater proportion of participants agreed that the benefits of antidepressants outweigh the risks (82.8%), taking antidepressants is relaxing (76.5%), and prevent breakdowns (75.3%). DAI scores ranged from − 8 to 10, with the majority of participants scoring above 0 (74.1%).

Significantly higher DAI scores (i.e., more positive attitudes towards antidepressants) were found among sixth year students (p-value = 0.001), students with higher family income (p-value = 0.001), and students with a personal history of using antidepressants (p-value < 0.001). Table 2 demonstrates the participants’ attitudes towards antidepressants.

**Table 2:** DAI responses of included participants

Statement	Type*	False N (%)	True N (%)
1. I think that the good things about antidepressants outweigh the bad	Positive	212 (17.2%)	1022 (82.8%)
2. If someone has to use antidepressants, they will feel strange, “doped up”, on them	Negative	680 (55.1%)	554 (44.9%)
3. If I need to take an antidepressant, I will take it of my own free well	Positive	651 (52.8%)	583 (47.2%)
4. If someone has to take antidepressants, it will make them feel more relaxed	Positive	290 (23.5%)	944 (76.5%)
5. If someone has to take antidepressants, it will make them feel tired and sluggish	Negative	788 (63.9%)	446 (36.1%)
6. If you have to take antidepressants, you will only take them when you feel ill	Negative	927 (75.1%)	307 (24.9%)
7. If someone has to take antidepressants, they will feel more normal on them	Positive	343 (27.8%)	891 (72.2%)
8. I think it is unnatural for someone’s mind and body to be controlled by antidepressants	Negative	760 (61.6%)	474 (38.4%)
9. I think those who take antidepressants have clearer thoughts on them	Positive	386 (31.3%)	848 (68.7%)
10. I think taking antidepressants will prevent those who take them from having a breakdown	Positive	305 (24.7%)	929 (75.3%)

*Type refers to the coding of the statement. Positive statements are “true” statements; thus, agreement responses are coded as 1. Negative statements are “false” statements; thus, agreement responses are coded as 0.

In terms of antidepressants knowledge, participants demonstrated an average understanding of antidepressants’ mechanisms of action. Contested facts included effect on monoamines, duration of the first course, and rate of remission of single agent therapy, as 39.2%, 42.0%, and 47.1% of all participants reported neutrality, respectively. Interestingly, 51.7% of students believed that antidepressants provide only a mere symptomatic treatment for depression. In terms of side effects, the majority of participants demonstrated acceptable knowledge of side effects. The association between initial usage of antidepressants and increased symptoms and the association between antidepressants and drug-drug interactions were also contested, as only 43.4% and 34.5% agreed with the aforementioned statements, respectively.

With regards to antidepressant use among special groups, the majority of the participants were unsure or falsely assured of the uses of antidepressants among pregnant women (70.2%), breastfeeding women (72.9%), and pediatrics (57.9%). Year of study (i.e., sixth year) (p-value < 0.001), higher GPA (p-value < 0.001), family income (i.e., 1000 + JOD) (p-value < 0.001), personal history of antidepressants (p-value < 0.001), family history of psychiatric illnesses (p-value < 0.001), active smokers (p-value < 0.001), and regular consumers of alcohol (p-value = 0.003) demonstrated significantly higher knowledge scores. Table 3 demonstrates the participants’ responses to knowledge items.

**Table 3:** Knowledge responses of included participants across different domains

Item	Disagree N (%)	Neutral N (%)	Agree N (%)
**Mechanism of action**			
1. Antidepressants are drugs that require continuous regular use, and are not used as “per needed”	168 (13.6%)	171 (13.9%)	895 (72.5%)
2. Antidepressants show their effects after a few weeks.	196 (15.9%)	304 (24.6%)	734 (59.5%)
3. All antidepressants aim to increase level of monoamines	455 (37.8%)	471 (39.2%)	277 (23.0%)
4. Everyone responds the same to a particular antidepressant	1014 (82.2%)	116 (9.4%)	104 (8.4%)
5. Antidepressants provide symptomatic treatment	191 (15.5%)	405 (32.8%)	638 (51.7%)
6. A period of 9–12 months is the typical duration of first course of antidepressants	200 (16.2%)	518 (42.0%)	516 (41.8%)
7. It takes at least 4 to 6 weeks at full dose before the antidepressant medication is deemed non-efficacious	170 (13.8%)	474 (38.4%)	590 (47.8%)
8. Treatment with a single antidepressant leads to remission in approximately half of patients	256 (20.7%)	581 (47.1%)	397 (32.2%)
**Side effects**			
9. Antidepressants are addictive	498 (40.4%)	327 (26.5%)	409 (33.1%)
10. Antidepressants are associated with sexual side effects (such as decreased libido, delayed ejaculation)	126 (10.2%)	293 (23.7%)	815 (66.0%)
11. Serotonin syndrome is a rare but serious complication of antidepressant use	94 (7.6%)	326 (26.4%)	814 (66.0%)
12. SSRIs have side effects of GI complaints	89 (7.2%)	316 (25.6%)	829 (67.2%)
13. Antidepressants discontinuation needs tapering due to associated withdrawal effects	101 (8.2%)	247 (20.0%)	886 (71.8%)
15. All antidepressants are associated with drug-drug interactions	310 (25.1%)	498 (40.4%)	426 (34.5%)
16. Serotonin syndrome can result from taking a single serotonergic agent or by taking multiple agents in combination	150 (12.2%)	499 (40.4%)	585 (47.4%)
**Usage in special groups**			
17. We can use antidepressants in pregnant women	382 (31.0%)	484 (39.2%)	368 (29.8%)
18. Antidepressants are safe during breastfeeding	351 (28.4%)	549 (44.5%)	334 (27.1%)
19. Antidepressants can’t be used in ages 0–6	267 (21.6%)	448 (36.3%)	519 (42.1%)
**Miscellaneous**			
20. It’s difficult to predict which patient will respond to which antidepressant, so trials of several antidepressants may be necessary before an effective agent is found	188 (15.2%)	321 (26.0%)	725 (58.8%)
21. MAOIs (monoamine oxidase inhibitors) are the first-line treatment for major depressive disorder	517 (41.9%)	432 (35.0%)	285 (23.1%)
22. SSRIs are the most common antidepressants in clinical use.	85 (6.9%)	298 (24.1%)	851 (69.0%)
23. There are modalities of depression treatment other than medications	103 (8.3%)	256 (20.7%)	875 (70.9%)

**Fig. 1:**
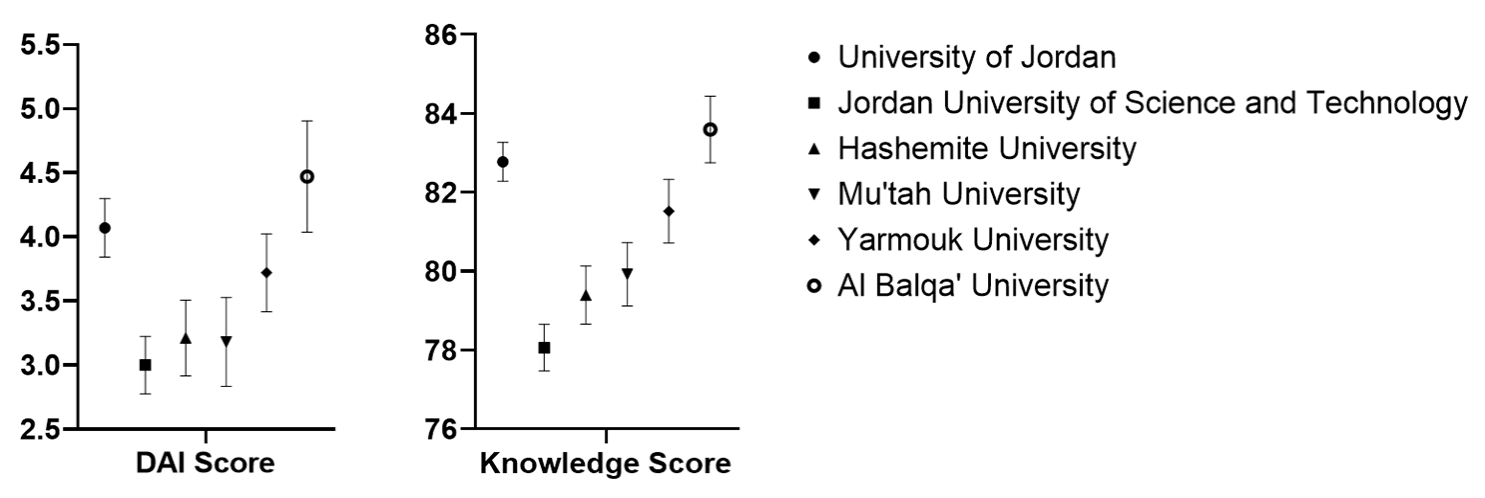
Knowledge and attitudes of included students stratified by medical school

With respect to differences in attitudes and knowledge scores among different universities, students from Al Balqa Applied University (p-value = 0.017) and the University of Jordan (p-value = 0.008) demonstrated significantly higher DAI scores than students from the Jordan University of Science and Technology. Additionally, significantly higher knowledge scores were found for students from Al Balqa Applied University and the University of Jordan compared to both their counterparts at the Jordan University of Science and Technology (p-value < 0.001 and < 0.001, respectively) and the Hashemite University (p-value = 0.002 and 0.008, respectively) (Refer to Fig. 1).

Table 4 demonstrates the predictors of knowledge scores. The multivariate linear regression model demonstrated that year of study (B: 2.928; 95%CI: 2.302–3.553), GPA (B: 2.458, 95%CI: 1.791–3.124), history of antidepressant use (B: 2.810; 95%CI: 1.335–4.286), family history of psychiatric disease (B: 1.312; 95%CI: 0.042–2.581), and DAI score (B: 0.514; 95%CI: 0.388–0.639) were positive predictors of knowledge scores. On the other hand, number of siblings (B: -0.328; 95%CI: -0.516 – -0.141) and studying at Jordan University of Science and Technology (B: -4.761: 95%CI: -6.795 – -2.727) were negative predictors of antidepressant knowledge.

**Table 4:** Predictors of antidepressant knowledge

Variable	B	SE_B_	t (B/SE_B_)	p-value	Lower 95%CI of B	Upper 95%CI of B
Female gender	0.688	0.545	1.264	0.207	-0.380	1.757
Year of study	2.928	0.319	9.181	**< 0.001**	2.302	3.553
GPA	2.458	0.340	7.235	**< 0.001**	1.791	3.124
Family income	0.239	0.374	0.639	0.523	-0.495	0.972
Number of siblings	-0.328	0.095	-3.441	**0.001**	-0.516	-0.141
History of using antidepressants	2.810	0.752	3.738	**< 0.001**	1.335	4.286
Family history of psychiatric treatment	1.312	0.647	2.027	**0.043**	0.042	2.581
DAI score	0.514	0.064	8.042	**< 0.001**	0.388	0.639
Occasional or regular alcohol consumption	1.278	1.177	1.086	0.278	-1.031	3.587
Active smoking	1.301	0.655	1.987	**0.047**	0.017	2.585
Attending Jordan University of Science and Technology*	-4.761	1.037	-4.593	**< 0.001**	-6.795	-2.727

B: Unstandardized regression coefficient; SE_B_: Standard Error of B; t: t-statistic

*Other universities were dummy coded into the model and their insignificant results are not present for ease of reporting.

## Discussion

The main results of our study identified the perspectives and knowledge of antidepressants among medical students. Our cohort generally displayed positive attitudes toward the medications, with almost two-thirds of the sample reporting positive views. Knowledge levels were acceptable, with some misconceptions regarding the mechanisms of action of antidepressants and their utilization in pregnant and breastfeeding women. Similar studies among healthcare undergraduates have shown varying results in these domains [17, 18, 26].

Considering that depression is the leading cause of disability worldwide [27], it is important to train healthcare professionals to correctly identify proper diagnostic and treatment methods. Factors influencing the provision of care to patients primarily pertain to their attitudes towards their health issues. Positive attitudes enable patients to access better services, improve their adherence, and augment the quality of their received care. In this context, studying these factors in medical students, the future gatekeepers of managing depression, is crucial considering their direct impact on the wellbeing of their patients [23].

One major aspect of decreasing stigmatizing attitudes among students is through enhancing education. As reflected in our results, students in the later years of their medical school and those with higher GPA have been more knowledgeable and have more positive attitudes toward antidepressants. The relationship between knowledge and attitude is bidirectional, in which an increase in one parameter will be positively correlated with the other and vice versa. This extends to depression and its treatment and aligns well with what other studies have reported, as they show a direct correlation between adequate knowledge of depression and antidepressants with more favorable attitudes towards medications [28–30].

Previous family history of psychiatric treatment served as a positive predictor for the increased knowledge scores about antidepressants. Even among students who are yet to complete their psychiatric training, social contact with mental health patients is an effective way of improving attitudes and reducing stigma, and hence, increasing knowledge regarding the conditions and treatment modalities [31].

Active smoking served as a positive predictor of antidepressant knowledge. Generally, the overlapping and alternating nature of substance use and mental health-related problems relates to the self-medication model. Numerous studies have been conducted, drawing associations of tobacco’s chemical properties as potential alleviators of some symptoms of mental illness, such as that of depression [32, 33]. The association becomes more evident as some researchers propose a causal relationship between poor mental health and smoking [34]. Such correlations extend to include other substances such as alcohol, as large epidemiologic studies showcase that self-medication practices with alcohol and/or other drugs is a common occurrence with up to 24% of individuals with anxiety and mood disorders using those substances to cope with their feelings of depression and anxiety [35, 36]. Some studies report lower levels of treatment seeking in patients with depression who use alcohol, which may further support the self-medication hypothesis [37].

Interestingly, a history of antidepressants use was associated with increased attitude and knowledge scores. Although mental health issues are quite common in the Arab world, significantly fewer people are treated for severe mental health illnesses than elsewhere globally [38, 39]. One of the most commonly reported factors affecting help-seeking attitudes toward mental illness is often related to stigma [13]. The professional culture of medicine not only reflects society by assigning stigma to people with mental health difficulties, but may also contribute to the high rates of suicide among healthcare professionals by delaying treatment seeking [40]. Adequate mental health for future doctors is crucial, as they will invariably confront highly stressful events as well as institutional pressure. Providing proper education and training on mental health problems and their management, including the appropriate utilization of psychotropic medications, is an important step in reducing the under-treatment of depression and improving students’ overall mental health and wellbeing [28].

A considerable number of students in our sample did not fully understand the utility of antidepressants for pregnant and breastfeeding women. Previous studies have shown the stigmatizing views of many medical students and healthcare professionals towards depression and a significant lack of knowledge about the use of antidepressants in perinatal depression [41]. These results prompt urgent changes to the current psychiatric educational curriculum. Depression is a prevailing issue in pregnancy, affecting at least one in seven women in the U.S [42, 43]. Evidence shows that perinatal depression increases the risk of many adverse events for both the mother and fetus [44]. Although SSRIs, the most commonly used antidepressants for depression, and other antidepressants cross the placental and fetal blood brain barrier, they generally have very safe profiles and minimal associated risks [45–47]. It should be noted, however, that extra considerations should be attended to when prescribing antidepressants to breastfeeding women. Although commonly prescribed drugs have a generally safe profile, newer agents and those with limited information regarding their use in breastfeeding, must be used with caution [48]. Efforts to educate medical students to properly identify and manage depression in this population are of utmost importance.

Some participants in our cohort held common misconceptions regarding antidepressants’ use. Firstly, almost 25% of the students agreed that antidepressants can only be taken when someone feels ill. In spite of the presence of strong evidence that suggests continuous use of antidepressants for almost 6 months [49], non-adherence or discontinuation remains a major obstacle to achieving good therapeutic outcomes. Although stigmatizing views towards depression have been reported to adversely affect adherence to medication [50, 51], their impact on Jordanian patients is still unclear. Secondly, about 36% of the students agreed that taking antidepressants would cause tiredness. Although fatigue is a relatively common side effect of antidepressants, especially in the early phases of treatment [52, 53], psychiatrists and other medical practitioners who prescribe antidepressants should be aware of the adverse effect profiles and educate patients accordingly in order to decrease morbidity and treatment discontinuation.

To remedy some of the misconceptions found in our results, we propose pedagogical approaches that may facilitate improvements in this regard. Psychiatric education remains the most effective way to improve knowledge and decrease stigma regarding mental disorders. Specifically tailored teaching with an emphasis on depression manifestations, causes, treatment modalities, and somatic reflections is likely to reduce misconceptions [31]. More precisely, linking the rationale for the use of antidepressants with that of underlying biological etiologies of mental disorders was shown to have increased acceptance for both, the conditions and their treatments, in the general public [54]. Improving communication skills between physicians and patients, specifically discussing the risk-to-benefit ratio of antidepressants, may also be relevant in this context as well [55].

Moreover, as our study highlights the interaction between knowledge of and attitude towards antidepressants, these results could be used to inform new educational activities that do not solely focus on increasing knowledge, but also address potential negative attitudes towards these medications. Incorporating a case-based learning experience that illustrates the benefits and risks of antidepressants in realistic clinical settings could be a possible approach. Interventions that lead to the de-stigmatization of mental health problems, particularly depression, are likely to be beneficial. Improving both theoretical and on-site education and enforcing them with in-depth learning in small groups could be a possible first step. Increasing social contact with mental health patients is an effective approach to reducing stigma. This is shown well in the literature in what is termed the ‘contact hypothesis’ [56]. Previously implemented programs advocating for optimistic attitudes when teaching about managing and treating individuals with mental disorders have shown impactful results in those regards as well.

The limitations of our study might affect the generalizability of our findings. These include non-randomized sampling technique, the use of a face-validated questionnaire, vulnerability to biases introduced by cross-sectional designs (e.g., social desirability bias), and the close-ended nature of questionnaire. Future research should attempt to create a holistic, psychometrically valid questionnaire that investigates knowledge of both depression and the variety of anti-depressant classes in a fashion tailored to medical students and primary care practitioners.

## Conclusion

In light of what is above, Jordanian medical students’ attitudes and knowledge regarding antidepressants, while acceptable, leave room for significant improvement. Yet, it appears that significant differences in both attitudes and knowledge exist across medical schools, which may indicate a gap in either training or teaching methodology. Therefore, current curriculums and clerkships could be revised as to encourage deeper student engagement in psychiatric training.

## Data Availability

The datasets used and/or analysed during the current study are available from the corresponding author on reasonable request.
